# A novel model of ambulatory teaching of residents in general practice in China: a cross-sectional study

**DOI:** 10.1186/s12909-024-05647-0

**Published:** 2024-06-19

**Authors:** Lingbo Liang, Xiangping Liu, Lin Zhang, Qiaoli Su

**Affiliations:** 1 General Practice Ward/International Medical Center Ward, General Practice Medical Center, West China Hospital, Sichuan University,, Chengdu, 610041 China; 2 Department of Primary Health Care, The fourth People’s hospital of Dazhu County, Dazhou, 635100 China

**Keywords:** Ambulatory teaching, General practice, General practitioner, Resident training

## Abstract

**Background:**

This study aims to determine the satisfaction and future training needs of general practice residents participating in a novel model of ambulatory teaching aligned with the specifications for standardized residency training in outpatient management issued by the Chinese Medical Doctor Association (CMDA).

**Methods:**

A cross-sectional survey of the satisfaction and training needs was conducted among general practice residents at West China Hospital, Sichuan University. Patient characteristics and preceptors’ feedback on the residents’ performance were also analyzed.

**Results:**

The study involved 109 residents (30.28% men) and 161 patients (34.78% men; age: 52.63 ± 15.87 years). Residents reported an overall satisfaction score of 4.28 ± 0.62 with the ambulatory teaching program. Notably, residents scored lower in the Subjective-Objective-Assessment-Plan (SOAP) evaluation when encountering patients with the greater the number of medical problems (*P* < 0.001). Residents encountering patients with a shorter duration of illness (< 3 months) achieved higher scores than those with longer illness durations (≥ 3 months, *P* = 0.044). Residency general practitioners (GPs) were most challenged by applying appropriate and effective patient referrals (43/109; 39.45%). GPs expressed a strong desire to learn how to make decisions when facing challenging patient situations (4.51 ± 0.63).

**Conclusion:**

This study suggests selecting patients with multiple comorbidities for ambulatory teaching and enhancing training on practical problem-solving abilities for GPs. The findings provide insights for the development of future ambulatory teaching programs.

**Supplementary Information:**

The online version contains supplementary material available at 10.1186/s12909-024-05647-0.

## Introduction

Outpatient consultation has long been regarded as one of the core competencies of general practitioners (GPs). Unlike inpatient treatment in the ward, treatment in the outpatient clinic requires GPs to build efficient and harmonious interaction with patients, and solve diverse and unpredictable problems in a short period of time [1]. Hence, training programs for outpatient teaching and ward teaching should differ in terms of both training patterns and training priorities. The “clinic first model” of general practice residency training aims to improve the quality of patient management and the participation of residents in ambulatory teaching programs [2–4]. According to the Chinese Medical Doctor Association (CMDA) [5], ambulatory teaching is a key part of standardized residency training that focuses on promoting the residents’ ability to independently manage outpatients under the supervision of preceptors. Ambulatory teaching should emphasize preceptor-resident interaction and aim to elevate the residents’ practical problem-solving and clinical thinking abilities [5].

Several observational research studies of Chinese GPs’ clinic consultations have reported insufficiencies in the following aspects of general practice: physical examination, management of multiple chronic diseases [6, 7], procedures, health promotion, informing family members, substance use counselling, and smoking cessation counselling [1]. Moreover, the current standardized training model for the ambulatory teaching of general practice in China lacks opportunities for the practical training of residents. In addition, the number of outpatients included in ambulatory teaching programs is limited; the disease spectrum involved is narrow; the training of clinical thinking is neglected; and the ability of independent and continuous management of outpatients is insufficient [8]. Therefore, ambulatory teaching in China should focus on strengthening the above components.

Recent studies have shown that to impart good-quality outpatient teaching, both the educational organization and preceptors should attach importance to procedures and clinic settings [9], strengthen the feedback and communication from preceptors to residents [10, 11], select appropriate patients for the training program, and increase patient engagement [11, 12]. Unfortunately, the published research on ambulatory teaching programs for general practice in China has mainly focused on the evaluation of residents’ performance [13, 14], and lacks assessments of the design of the teaching program itself and preceptor-resident interaction [15, 16]. To improve the quality of ambulatory teaching programs for general practice in China, the CMDA, in July 2021, organized experts to compile specifications for standardized residency training for outpatient management in general practice [5]. These specifications expound in detail the procedure and implementation of ambulatory teaching, and emphasize preceptor-resident interaction and the elevation of residents’ physical examination, practical problem-solving, and clinical thinking abilities; they provide specific practical norms for the ambulatory teaching of general practice, and make up for the shortcomings in the previous literature on ambulatory teaching [9, 17]. However, to date, no study has analyzed the implementation of ambulatory teaching programs based on the above specifications. This study aims to determine the satisfaction and future training needs of general practice residents participating in a novel model of ambulatory teaching aligned with the specifications for standardized residency training in outpatient management issued by the CMDA.

## Participants and methods

### Study design and study setting

A novel model of ambulatory teaching utilized in this study aligns with the regulations and guidelines set forth by the CMDA in 2021, and this model represents the standardized residency training for outpatient management in general practice endorsed by the CMDA. The ambulatory teaching program is scheduled to take place on every Wednesday in the General Practice Medical Center of West China Hospital. According to the CMDA guidelines, the preceptors of the outpatient teaching program should be GPs who have held an intermediate professional title for at least 3 years or GPs who hold a senior professional title; additionally, they should be qualified by the national or provincial faculty for training in general practice (completed ≥ 56 h of total training time and passed an examination).

**Fig. 1:**
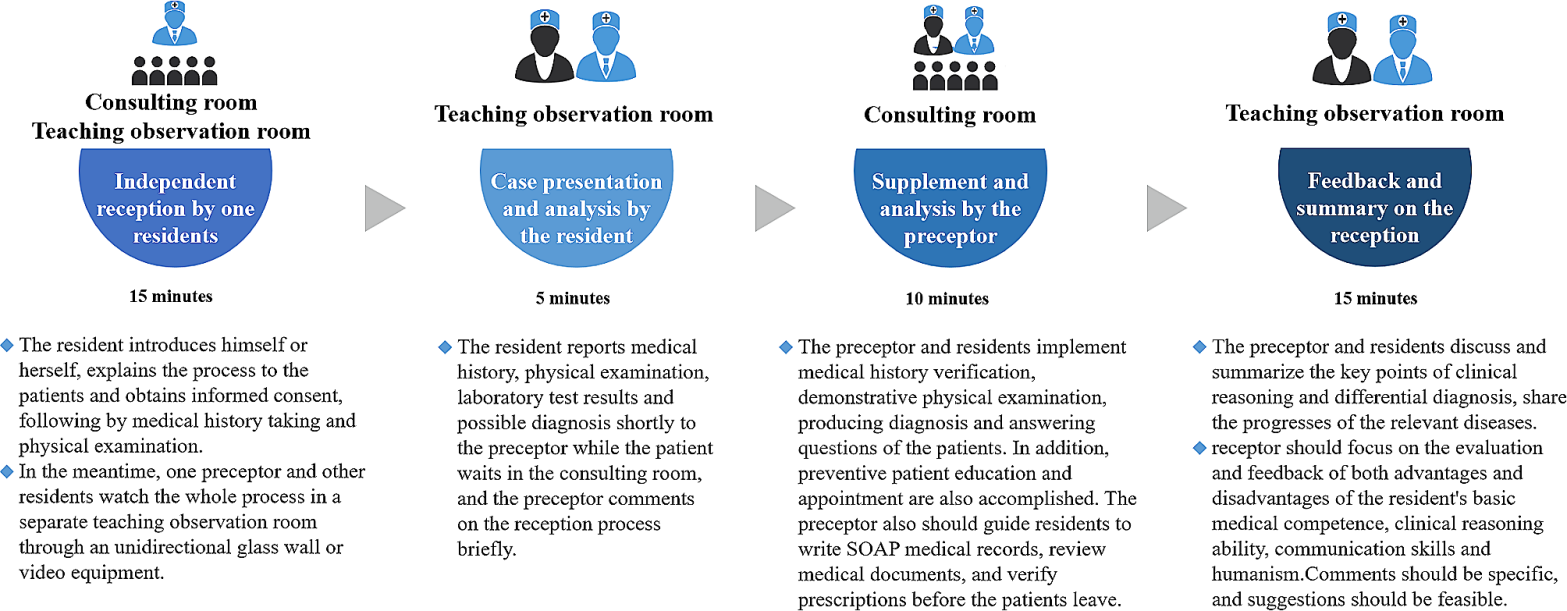
The procedure, clinic setting, duration, and main contents of each part of the ambulatory teaching program

The principles of the CMDA guidelines can be summarized as follows: (1) the process of meeting and examining the patient is primarily performed by the resident; (2) in addition to independent patient consultations by the residents, the training program emphasizes the interaction between preceptors and residents; (3) real-world patient consultations form the main teaching contents; and (4) specific feedback to the residents by the preceptor is crucial. The procedure, clinic setting, duration, and main contents of each part of the ambulatory teaching program are summarized in Fig. 1.

### Data collection

The information of the patients who participated in the ambulatory teaching program between 27 February 2023 and 31 August 2023 was extracted through the hospital information system of West China Hospital of Sichuan University. We collected the patients’ demographic information, reasons for encounter (RFEs), comorbidities, hospital costs, and prescriptions as well as the referral advice given to the patients and data on their previous visits to the hospital. The demographic characteristics of the residents who participated in the ambulatory teaching program were also collected.

### Patient recruitment and informed consent

The inclusion criteria were as follows: (1) patients referred from primary healthcare institutions, (2) patients with complex chronic conditions requiring comprehensive evaluation and guidance, (3) patients with symptoms as the main manifestations (excluding critical conditions), and (4) patients who were willing to participate in the ambulatory teaching program. The following exclusion criteria were applied: (1) patients in critical condition requiring emergency treatment, (2) patients who refused to participate in the ambulatory teaching program, and (3) patients whose family members visited the clinic instead of the patients themselves.

All recruited patients received a brief introduction to the ambulatory teaching program at the time of online or local registration, so that they could agree or decline to participate in the program. A written informed consent form was offered to each patient by the resident before the outpatient consultation. The resident also clarified that desensitized data would be collected from the patient’s medical records, and their interactions with the doctors would be videotaped for educational and scientific purposes only.

### Patient characteristics

A total of 161 patients participated in this research study between 27 February 2023 and 31 May 2023. The demographic information of the participating patients is shown in Table 1. The average age of the patients was 52.63 ± 15.87 years (range: 18–88 years). The average duration of consultation was 13.16 ± 4.10 min (range: 5–23 min). The median cost of the outpatient consultation was 193 RMBs (range: 22–1102 RMBs). Of the 161 patients, 45.96% were visiting for the first time (first consultation), and 54.04% were revisiting (follow-up consultation). Nearly half of the patients who visited the ambulatory teaching clinic complained of a single health problem; 18.01% of the patients reported 2 problems, while 43.3% of the patients had 3 or more problems. The majority of the patients (72.67%) had experienced their current health issue for longer than 3 months.

**Table 1:** Patients’ characteristic and visits’ characteristics

Variables	Number (*n*)	Percentage (%)
Gender (*n* = 161)		
Male	56	34.78
Female	105	65.22
Age (years) (*n* = 161)		
< 45	49	30.43
45–65	76	47.20
> 65	36	22.36
Occupation (*n* = 161)		
Employed	68	42.23
Retired	66	40.99
Unemployed	27	16.77
Marital status (*n* = 161)		
Unmarried	11	6.83
Married	144	89.44
Divorced or widowed	6	3.73
Whether patients have visited department of general practice before the ambulatory teaching (*n* = 161)		
Yes	54	33.54
No	107	66.46
Types of visits (*n* = 161)		
First visit for current health issue	74	45.96
Revisit for current health issue	87	54.04
Number of medical problems discussed in the ambulatory teaching (*n* = 161)		
1	80	49.70
2	29	18.01
3 or more	52	32.30
Duration of current health issue (*n* = 161)		
< 3 months	44	27.33
≥ 3 months	117	72.67

### Satisfaction questionnaire

A questionnaire to assess the residents’ satisfaction with the ambulatory teaching program was designed based on the Leicester assessment package [18, 19], Maastricht History-taking and Advice Scoring Global list [20], expert assessments, and one-round of pre-investigation. All items in the questionnaire were classified into 3 categories by principal component analysis: (1) process and clinic setting, (2) patient characteristics, and (3) preceptor behaviors. The questions were self-evaluated using a 5-point Likert scale anchored with terms for agreement (1: strongly disagree, 2: disagree, 3: neutral, 4: agree, and 5: strongly agree). The satisfaction scales of ambulatory teaching of residents and the training needs of ambulatory teaching of residents were shown in Supplementary files 1–2.

### Preceptors’ feedback on the residents’ performance

Instant feedback on the performance of each resident was given and recorded by the preceptor during the fourth section of the ambulatory teaching program. After receiving the feedback, all participating residents were asked to submit a medical record of a patient he or she treated, in the form of a Subjective-Objective-Assessment-Plan (SOAP). Each SOAP record was scored by the same preceptor based on the Guidelines for SOAP Oral Case Presentation Assessment in Standardized Residency Training (2022 edition) [21]. The first part of the SOAP form included sub-item evaluations of the subjective Sect. (6 items), objective Sect. (6 items), assessment Sect. (4 items), and plan Sect. (4 items). Each item was graded as “not applicable,” “content completely missing,” “content partially missing,” or “content complete.” The second part of the SOAP form was an overall evaluation using a 5-point scale based on the comprehensiveness of information gathering, listing of health issues, diagnosis and treatment plan, organization, communication skill, and professional quality. Overall SOAP scores of 1 to 2 indicated that the resident’s performance did not meet requirements; a score of 3 indicated that the resident’s performance meets requirements, and scores of 4 to 5 indicated excellent performance (details shown in Supplementary files 3–4).

### Training needs of residents

The training needs of the participating residents were identified through a cross-sectional survey. The questionnaire was designed based on recently published data on the characteristics of outpatients in general practice [1, 22, 23], previous research on ambulatory teaching [24, 25], expert assessments, and one round of pre-investigation. All items in the survey questionnaire were classified into 4 categories by principal component analysis (Supplementary Material 2): (1) greeting and history taking, (2) physical examination, (3) problem solving, and (4) anticipatory care. The questionnaire items were self-evaluated using a 5-point Likert scale (1: strongly disapprove, 2: disapprove, 3: neutral, 4: approve, and 5: strongly approve).

### Statistical analysis

All data from both questionnaires were exported from the questionnaire network (www.wjx.cn). The internal consistency of the satisfaction questionnaire and the training needs questionnaire was assessed using the Cronbach *α* coefficient. The Kaiser–Meyer–Olkin (KMO) score and the significance of the Bartlett test of sphericity were reported. The Kolmogorov–Smirnov test was used to test the assumption of normal distribution. The Mann–Whitney *U* test was used for comparisons between 2 rank variables, and the Kruskal-Wallis *H* test was used for comparisons among more than 2 rank variables. The scores for the 3 dimensions of residents’ satisfaction questionnaire and the overall SOAP evaluation score were presented as mean and standard deviation. All statistical analyses were performed using SPSS version 22.0 (SPSS Inc., Chicago, IL, USA). A *P*-value < 0.05 was considered statistically significant.

## Results

### Demographic data and satisfaction scores of the residents

In total, 109 residents participated in the ambulatory teaching program in the General Practice Medical Center of West China Hospital, Sichuan University during the study period. They all responded to the satisfaction questionnaire without any missing information or misinformation (response rate, 100%). The Cronbach α coefficient of the satisfaction questionnaire was 0.978, whereas its KMO score was 0.915. The Bartlett test of sphericity was significant (*P* < 0.05).

Table 2 summarizes the correlation of demographic variables with the satisfaction scores of the residents. We found that 30.28% of the residents were male, and the highest education level of most of the residents was a bachelor’s degree (93.58%). The majority of the participants (72.48%) had passed the National Medical Licensing Examination. Only 10 residents (9.17%) had never participated in any form of outpatient clinic training before this ambulatory teaching program.

**Table 2:** Correlation of demographic variables of residents with satisfaction scores of outpatient teaching program

Variables	Number, Percentage (%)	Process and clinic setting			Patient characteristics			Preceptor behaviors		
		Score	Z/H values	*P*	Score	Z/H values	*P*	Score	Z/H values	*P*
Gender			-1.42	0.16		-0.88	0.38		-0.84	0.40
Male	33 (30.28)	4.17 ± 0.58			3.97 ± 0.76			4.38 ± 0.63		
Female	76 (69.72)	4.31 ± 0.69			4.14 ± 0.70			4.45 ± 0.64		
Educational background			-1.45	0.15		-0.71	0.48		-1.42	0.15
Bachelor	102 (93.58)	4.25 ± 0.66			4.06 ± 0.71			4.40 ± 0.64		
Master	7 (6.42)	4.54 ± 0.74			4.26 ± 0.63			4.77 ± 0.43		
Years of standardized training of residents			6.81^†^	0.08		3.11^†^	0.38		8.80^†^	0.08
First year	27 (24.77)	4.01 ± 0.72			3.89 ± 0.74			4.17 ± 0.70		
Second year	31 (28.44)	4.24 ± 0.65			4.12 ± 0.69			4.42 ± 0.64		
Third year	41 (37.61)	4.37 ± 0.64			4.10 ± 0.70			4.53 ± 0.59		
Specialty training stage	10 (9.17)	4.57 ± 0.43			4.26 ± 0.78			4.70 ± 0.48		
Whether or not pass the National Medical Licensing Examination			-2.82	**0.005**		-2.32	*0.021*		-2.80	**0.005**
Yes	79 (72.48)	4.37 ± 0.61			4.17 ± 0.70			4.53 ± 0.58		
No	30 (27.52)	3.97 ± 0.71			3.80 ± 0.68			4.15 ± 0.70		
Forms of outpatient clinic training before participating in this ambulatory teaching			3.67^†^	0.16		1.11^†^	0.57		2.06^†^	0.36
Have not attended any form of outpatient clinic training	10 (9.17)	4.17 ± 0.78			4.05 ± 0.67			4.44 ± 0.71		
Participate in outpatient clinic practice under supervision	62 (56.88)	4.19 ± 0.65			4.03 ± 0.66			4.35 ± 0.65		
Independently practice outpatient clinic medical service	37 (33.94)	4.42 ± 0.64			4.14 ± 0.80			4.54 ± 0.59		
Times of ambulatory teaching residents have attended			0.76^†^	0.68		2.53^†^	0.29		0.64^†^	0.73
1 time	56 (51.38)	4.25 ± 0.64			4.00 ± 0.69			4.42 ± 0.62		
2 times	28 (25.69)	4.19 ± 0.78			4.09 ± 0.76			4.36 ± 0.72		
≥ 3 times	25 (22.94)	4.38 ± 0.56			4.26 ± 0.72			4.51 ± 0.59		

^†^H value

The overall satisfaction score of the residents with the ambulatory teaching program was 4.28 ± 0.62. The satisfaction scores for the dimensions of process and clinic settings, patient characteristics, and preceptor behaviors were 4.26 ± 0.66, 4.07 ± 0.71, and 4.43 ± 0.61, respectively. We found that the satisfaction score did not significantly differ with gender (male vs. female), educational background (bachelor’s vs. master’s degree), year of standardized residency training in general practice (first year vs. second year vs. third year vs. specialty training stage), number of times the residents attended ambulatory teaching (1 time vs. 2 times vs. ≥3 times), forms of outpatient clinic training before participating in our ambulatory teaching program (did not attend any form of outpatient learning vs. participated in outpatient practice under supervision vs. independently practiced outpatient medical service; all *P* > 0.05). However, all 3 satisfaction subscale scores of residents who had passed the National Medical Licensing Examination were significantly higher than those of residents who had not passed this examination (all *P* < 0.05).

### Patients’ RFEs and GPs’ prescriptions

There were 362 RFEs (2.24 per encounter) during our observation. The distribution of the RFEs is shown in Table 3. The top 10 most frequent RFEs were hypertension, pulmonary nodule, gastritis/gastroesophageal reflux disease, thyroid nodule, diabetes mellitus, dyslipidemia, neck pain and lower back pain, abdominal pain or distension, joint and muscle pain, and anxiety or depression. The top 10 most frequently issued auxiliary examinations/laboratory tests and the top 10 most frequently prescribed medicines are listed in Table 4.

**Table 3:** Summarization of patient reasons for encounter

Reasons for encounter	Frequency	Percentage (%)
Hypertension	32	19.88
Pulmonary nodule	24	14.91
Gastritis/gastroesophageal reflux disease	23	14.91
Thyroid nodule	22	13.66
Diabetes mellitus	18	11.18
Dyslipidemia	18	11.18
Neck pain and lower back pain	16	9.94
Abdominal pain or distension	16	9.94
Joint and muscle pain	14	8.70
Anxiety or depression	13	8.07
Breast nodules	12	7.45
Insomnia	12	7.45
Nasopharyngeal discomfort	9	5.59
Health consultation	8	4.70
Nephrolith	8	4.70
Malignant diseases	8	4.70
Osteoporosis	7	4.35
Cerebral infarction	7	4.35
Abnormal stool	6	3.73
Chest distress	6	3.73
Steatohepatitis	6	3.73
Fatigue	6	3.73
Erythra/pruritus	6	3.73
Subcutaneous nodule	6	3.73
Gallstone	5	3.11
Coronary heart disease	5	3.11
Atherosclerotic plaques of the carotid artery	5	3.11
Chronic obstructive pulmonary disease	5	3.11
Chest pain	4	2.48
Edema of lower extremities	4	2.48
Hyperuricemia	4	2.48
Chronic hepatitis B	4	2.48
Menstrual disorder	4	2.48
Loss of weight	3	3.73
Obesity	3	1.86
Arhythmia	3	3.73
Cough	3	1.86
Parkinson’s disease	3	1.86
Dryness in the mouth	2	1.24
Asthma	2	1.24

**Table 4:** The most frequently issued auxiliary examinations or laboratory tests and the most frequently prescribed medicine of the outpatient teaching

The most frequently issued auxiliary examinations or laboratory tests	Frequency	Percentage (%)	The most frequently prescribed medicine of the outpatient teaching	Frequency	Percentage (%)
Blood routine examination and biochemistry tests	55	34.16	Calcium channel blocker	28	17.39
Electrocardiograph	51	31.68	Atorvastatin/rosuvastatin	24	14.91
Ambulatory blood pressure	35	21.74	Angiotensin-converting enzyme inhibitor/Angiotensin receptor blockers	20	12.42
Ultrasonic cardiogram	34	21.12	Proton pump inhibitor/Gastric mucosal protective. agent/Compound digestive enzyme capsules	13	8.07
Thyroid function	34	21.12	Metformin hydrochloride	12	7.45
Carotid artery ultrasound	28	17.39	Insulin	8	4.97
Thyroid ultrasound	25	15.53	Alprazolam/Estazolam/Zolpidem tartrate/zopiclone	7	4.35
Chest CT scan	22	13.66	Repaglinide/Gliclazide/Migltol	7	4.35
Glycosylated hemoglobin	22	13.66	Alendronate sodiumand vitamin D3 tablets	6	3.73
Urinary albumin to creatinine ratio	21	13.04	Calcitriol	6	3.73

### Instant feedback by preceptors and SOAP records of residents

Five preceptors were included in this study; among them, two held senior professional titles, and three held intermediate professional titles. Analysis of the instant feedback on the performance of the 109 residents showed that the top 10 items commented on by the preceptors were as follows: lacking in skills of applying appropriate and efficient referral of patients (43/109, 39.45%), lacking in skills of recommending suitable complementary or alternative treatments to patients (36/109, 33.03%), lacking in skills of physical examination of key parts (35/109, 32.11%), lacking in skills of using a safe diagnostic strategy with first-visit patients (28/109, 25.69%), lacking in skills of making harmless decisions when temporarily unable to solve patients’ problems (28/109, 25.69%), lacking in well-organized approach to information gathering (26/109, 23.85%), lacking in normative management of pulmonary nodules (19/109, 17.43%), lacking in integrative prevention of arteriosclerotic cardiovascular disease (19/109, 17.43%), lacking in normative management of hypertension (14/109, 12.84%), and lacking in normative management of diabetes mellitus (14/109, 12.84%).

The overall evaluation of the SOAP records showed that 4.6% (*n* = 5) of the residents had acquired a grade 1 assessment, and 24.8% (*n* = 27) had acquired a grade 2 assessment (grades 1 to 2: does not meet requirements). In addition, 34.9% (*n* = 38) and 22.9% (*n* = 25) of the residents acquired grade 3 and grade 4 assessments, respectively (grades 3 to 4: meets requirements). Finally, 12.8% (*n* = 14) of the residents acquired a grade 5 assessment (grade 5: excellent performance). A longer training time of the residents was associated with higher overall SOAP scores (*P* < 0.001), and residents who passed the National Medical Licensing Examination achieved higher overall SOAP scores than residents who did not pass this examination (*P* = 0.011; Table 5). The greater the number of medical problems of each patient, the lower the residents’ overall SOAP score (*P* < 0.001). Residents who received patients with a duration of current health issue of < 3 months scored higher than residents who received patients with a duration of current health issue of > 3 months (*P* = 0.044).

**Table 5:** Correlation of demographic variables of residents and patients’ characteristics with overall SOAP score

Variables of residents and patients	Number, Percentage (%)	Overall evaluation of SOAP records		
		SOAP score	Z/H values	*P*
Residents’ characteristics				
Years of standardized training of residents			18.81^a^	**< 0.001**
First year	27 (24.77)	2.81 ± 1.03		
Second year	31 (28.44)	2.85 ± 1.08		
Third year	41 (37.61)	3.29 ± 0.96		
Specialty training stage	10 (9.17)	4.40 ± 0.70		
Whether or not pass the National Medical Licensing Examination			-2.54	**0.011**
Yes	79 (72.48)	3.30 ± 1.10		
No	30 (27.52)	2.73 ± 0.91		
Forms of outpatient clinic training before participating in this ambulatory teaching			3.384	0.184
Have not attended any form of outpatient clinic training	10 (9.17)	2.80 ± 0.92		
Participate in outpatient clinic practice under supervision	62 (56.88)	3.05 ± 1.05		
Independently practice outpatient clinic medical service	37 (33.94)	3.41 ± 1.14		
Times of ambulatory teaching residents have attended			4.825	0.09
1 time	56 (51.38)	3.09 ± 1.03		
2 times	28 (25.69)	2.93 ± 1.25		
≥ 3 times	25 (22.94)	3.52 ± 0.92		
Patients’ characteristics				
Whether have visited department of general practice before the ambulatory teaching			-0.667	0.505
Yes	35 (32.11)	3.09 ± 1.01		
No	74 (67.89)	3.18 ± 0.92		
Types of visits			-1.639	0.101
First visit for current health issue	56 (51.38)	3.30 ± 1.08		
Revisit for current health issue	53 (48.62)	2.98 ± 1.07		
Number of medical problems discussed in the ambulatory teaching			15.454	**< 0.001**
1	55 (50.46)	3.55 ± 1.00		
2	18 (16.51)	2.89 ± 1.13		
3 or more	36 (33.03)	2.67 ± 0.96		
Duration of current health issue			-2.012	**0.044**
< 3 months	25 (22.94)	3.52 ± 1.00		
≥ 3 months	84 (77.06)	3.04 ± 1.08		

^†^ H value

In the sub-item evaluations of the SOAP records, the top 5 items that were most frequently recorded as “content completely missing” were as follows: “time of next follow-up and review indicators” (49/109, 44.95%), “treatment expectation and patient education” (35/109, 32.11%), “auxiliary examination findings related to differential diagnosis” (31/109, 28.44%), “time of next follow-up and review index needed” (28/109, 25.69%), and “related negative signs” (23/109, 21.10%). The top 5 items most frequently recorded as “content partially missing” were as follows: “clinically significant negative symptoms” (27/109, 24.77%), “defining characteristics of differential diagnosis” (21/109, 19.27%), “heart and lung examination” (17/109, 15.60%), “disease development and treatment during the process” (14/109, 12.84%), and “physical examination of key parts and major positive signs” (10/109, 9.17%).

### Training needs of residents

To improve the quality of the ambulatory teaching program in the future, we conducted a survey of the training needs of residents in terms of the key contents of the ambulatory teaching program for general practice residents. The Cronbach α coefficient of the survey questionnaire was 0.984, and its KMO value was 0.919. The Bartlett test of sphericity was significant (*P* < 0.05). The average scores for each item of the survey questionnaire are shown in Table 6. The top 10 items with the highest scores were as follows: (1) learn how to make decisions that are harmless to patients when GPs are temporarily unable to solve patients’ problems (4.51 ± 0.63), (2) learn the most frequently prescribed medicines in outpatient clinics in general practice and the adverse effects of these medicines (4.50 ± 0.66), (3) learn the most frequently issued auxiliary examinations/laboratory tests for outpatients in general practice (4.49 ± 0.66), (4) learn the most common alternative therapies in the outpatient clinic in general practice (4.48 ± 0.63), (5) quick physical examination of shoulder and neck pain (4.48 ± 0.66), (6) learn the most common major health issues of outpatients in general practice (4.47 ± 0.66), (7) quick physical examination of the nervous system (4.47 ± 0.65), (8) learn to develop reasonable follow-up plans and achieve consensus for patients (4.46 ± 0.65), (9) quick physical examination of lower back pain (4.45 ± 0.66), and (10) phrase questions simply and clearly (4.45 ± 0.74).

**Table 6:** Item and dimension scores of the training needs questionnaire rated by residents

Item	Average score
*Part 1: Greeting and history taking*	
Learn the most common major health issues of outpatients of general practice	4.47 ± 0.66
Identifies patients’ reasons for consultation	4.42 ± 0.71
Seeks clarification of words used by patients as appropriate	4.42 ± 0.76
Phrases questions simply and clearly	4.45 ± 0.74
Recognizes patients’ verbal and non-verbal cues	4.31 ± 0.79
Exhibits well-organized approach to information gathering	4.41 ± 0.68
Considers physical, social and psychological factors as appropriate	4.33 ± 0.73
Makes, when necessary, proper confrontations or compromise	4.36 ± 0.75
*Part 2: Physical examination*	
Learn the palpation of the superficial lymph node	4.34 ± 0.75
Learn the palpation of the thyroid and breast nodules	4.37 ± 0.72
Quick physical examination of neural system	4.47 ± 0.65
Quick physical examination of shoulder and neck pain	4.48 ± 0.66
Quick physical examination of lower back pain	4.45 ± 0.66
Identification of common skin diseases in outpatient clinics	4.38 ± 0.72
*Part 3: Problem solving*	
Learn the disease spectrum of outpatients of general practice	4.40 ± 0.67
Learn the most frequently issued auxiliary examinations/laboratory tests in the outpatients of general practice	4.49 ± 0.66
Learn the most frequently prescribed medicine and adverse effects of theses medicines of the outpatient teaching	4.50 ± 0.66
Learn the cautions of issuing diagnosis certificate in outpatient clinic of general practice	4.41 ± 0.66
Learn the most common alternative therapies in outpatient clinic of general practice	4.48 ± 0.63
Learn how to make decisions that are harmless to patients when GPs are temporarily unable to solve patients’ problems	4.51 ± 0.63
Learn how to checks whether patient has understood information of therapies and follow-up arrangements	4.44 ± 0.66
Learn to develop reasonable follow-up plans and achieve consensus for patients	4.46 ± 0.65
*Part 4: Anticipatory care*	
Learn how to find appropriate opportunities for health promotion and disease prevention	4.42 ± 0.64
Learn the latest screening methods and progress in diagnosis and treatment of common diseases of outpatient of general practice	4.44 ± 0.64
Learn the cautions of vaccination	4.38 ± 0.68
Learn how to deal with the patient’s sleep disorders	4.42 ± 0.66
Learn how to give advice on exercise to outpatients	4.41 ± 0.68
Learn how to give advice on healthy diet to outpatients	4.39 ± 0.67
Learn how to help outpatients quit smoking	4.42 ± 0.66
Learn how to deal with a patient’s psychological counseling	4.42 ± 0.66

## Discussion

In China, the development of general practice residency training is still in its nascent stages, leading to a deficiency in ambulatory training for general practitioners. Existing training primarily relies on internships and observational learning, with limited opportunities for hands-on experience. Only in the third year of residency GPs begin to independently manage consultations, often with a narrow scope of diseases. This lack of comprehensive clinical training hampers their ability to handle diverse clinical scenarios effectively [8, 26].

Indeed, existing models such as modified OMP, SNAPPSS and MINI-CEX were used under different conditions [27–29]. Our model represents the comprehensive framework endorsed by the CDMA. More importantly, our model provides more detailed and practical guidance for standardized procedures in consultation rooms, teaching evaluation rooms, time management, and patient selection with drawing inspiration from existing models [27–29]. Our model aims to facilitate the implementation of general practice ambulatory teaching across hospitals in China and other countries, particularly those just beginning to offer such services. It is worth noting that patients are treated as authentic patients, providing real-world experiences for residents of GP in our teaching mode. They are not utilized as standardized patients, ensuring the authenticity and relevance of the training experience. This study has revealed that the ambulatory teaching program was generally well-received by residents, indicating their overall satisfaction with this innovative model of ambulatory teaching.

**Fig. 2:**
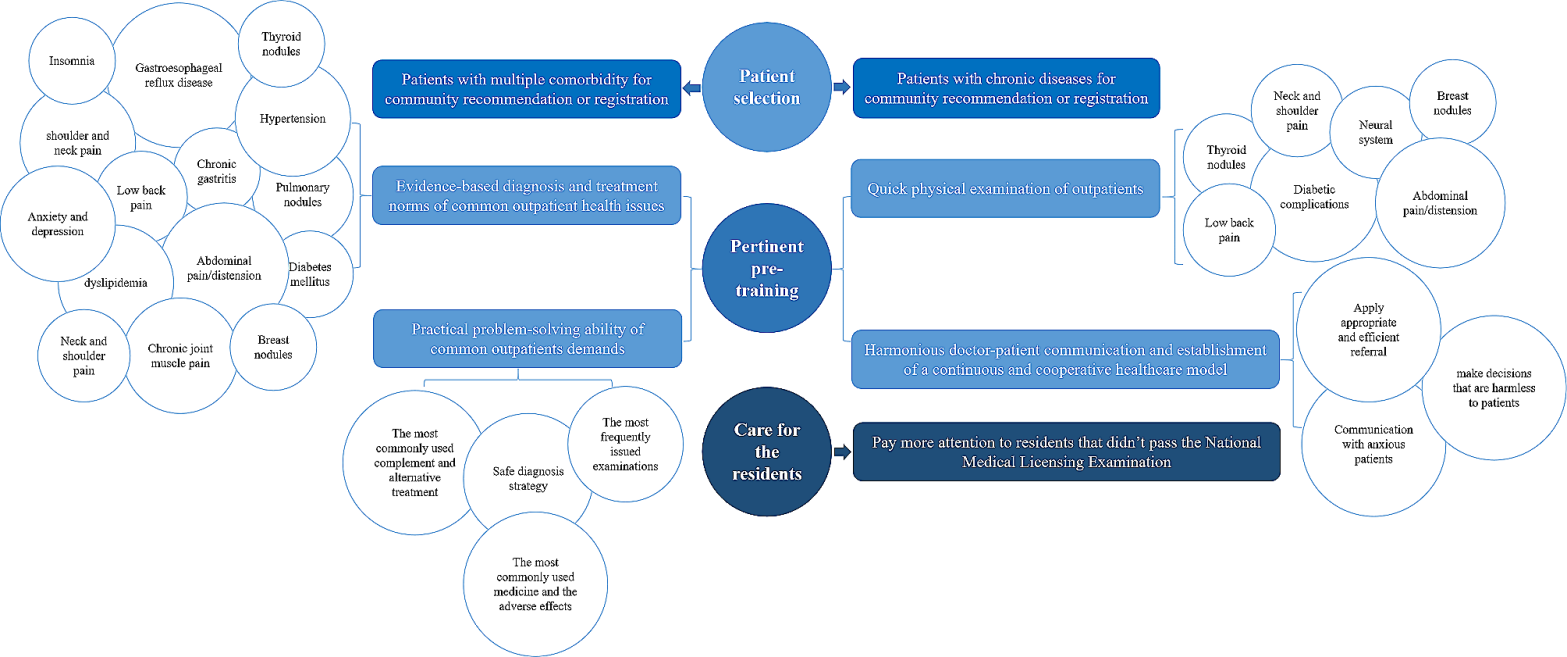
The hypothesis and structure of optimized ambulatory teaching

According to the results of this research study, we formulated recommendations to optimize ambulatory teaching programs for general practice residents in the future. First, the satisfaction score for patient characteristics was lower than the scores for process and clinic settings, and preceptor behaviors, so we recommend that future ambulatory teaching programs place more emphasis on patient selection. The management of patients with comorbidities or a long disease duration was a general weakness of the residents, according to the feedback of the preceptors and the SOAP evaluations. In addition, “lacking in the skill of applying a safe diagnostic strategy to first-visit patients” was a common feedback provided by the preceptors. Therefore, future ambulatory teaching programs should preferentially select revisiting patients with chronic comorbidities. Second, both preceptors and residents should receive pre-training centered on the common health issues identified in our research study (Fig. 2). The curriculum of this pre-training should comprise the following aspects: (1) evidence-based diagnosis and treatment of common outpatient health issues, (2) quick physical examination of outpatients, (3) practical problem-solving ability to manage common demands of outpatients, and (4) harmonious doctor-patient communication and establishment of a continuous and cooperative health care relationship. Third, preceptors should pay more attention to residents who have not passed the National Medical Licensing Examination since the average satisfaction score of these residents was lower than that of residents who did pass this examination.

Notably, our study revealed that residents scored lower in the SOAP evaluation when encountering patients with the greater the number of medical problems (*P* < 0.001). Residents encountering patients with a shorter duration of illness (< 3 months) achieved higher scores than those with longer illness durations (≥ 3 months, *P* = 0.044). These findings resonate with several studies highlighting the challenges faced by Chinese general practitioners in managing patients with complex medical conditions [7–30]. The reasons behind these results are likely multifaceted, influenced by factors such as workload [30], complexity of cases [1, 31], time constraints [1, 32], and the emotional impact of managing chronic conditions [1, 12, 32]. These factors may collectively contribute to the observed decrease in residents’ performance scores when faced with patients presenting with multiple medical problems or chronic health issues.

Additionally, several studies have explored residents’ preferences regarding independent patient encounters in the consultation room [33, 34]. Some advocate for ambulatory teaching clinics to be structured differently from conventional outpatient settings, often requiring specialized groups. Meanwhile, other studies have echoed our findings, suggesting that preceptors need not be physically present in the room but instead monitor residents’ performance via video. This approach fosters a sense of independence in decision-making while providing reassurance that preceptors are available to offer feedback [31, 35]. Notably, there remains a lack of detailed guidance on when preceptors should intervene without undermining residents’ sense of autonomy in patient care.

Our ambulatory teaching model addresses these concerns by addressing deficiencies in resident GPs’ reception skills within the outpatient department. By ensuring that patients receive quality medical treatment despite the residents’ varying levels of experience, our model also safeguards the authority of resident GPs during patient encounters. This approach fosters confidence and proactive engagement among resident GPs in independently managing patient care. While some studies advocate for residents and preceptors to prepare in advance by reviewing patient medical history and discussing key health issues [35]. Instead, our teaching mode diverges from this approach, and our model prioritizes the development of residents’ ability to respond to unexpected events and adapt to changes in patient conditions: a fundamental requirement for competent general practitioners.

### Strengths and implications of this study

This study summarized the characteristics of the patients included in the ambulatory teaching program for general practice residents, the satisfaction scores of the residents with the teaching program, and the feedback of the preceptors on the residents’ performance based on the CMDA-issued guidelines. This study has revealed that the ambulatory teaching program was generally well-received by residents, indicating their overall satisfaction with this innovative model of ambulatory teaching. We hope that the results of this study will help overcome the shortcomings of the specifications for standardized residency training for outpatient management in general practice, and provide a reference to improve the quality of ambulatory teaching programs for general practice in China and other countries, particularly those just beginning to offer such services.

### Limitations

The limitations of this research should be acknowledged: (1) The study lacks patient evaluation of ambulatory teaching. We considered that offering a satisfaction survey to patients during their visit may worry the patients and thus affect the authenticity of the results; therefore, we did not include a patient satisfaction survey in this study. (2) The residents’ satisfaction evaluations of the preceptors may be inflated based on concerns that low satisfaction scores would affect the preceptors’ perception of themselves. To overcome this deficiency, we will conduct a supplementary study in which a third-party participant evaluates the behavior and teaching quality of the preceptors. (3) The sample size was relatively small. In the future, we plan to improve the ambulatory teaching program with a digital auxiliary teaching system, and extend the program to the community. In this manner, we plan to expand the sample sizes of both residents and patients for further research to enrich our findings. (4) A high Cronbach’s alpha may indicate redundancy among the scale items, suggesting that some items may be similar. To reduce the Cronbach’s alpha value, we will undertake the following steps: (i) review each item in the scale to identify overlapping items and eliminate redundant ones; (ii) consider modifications to the scale items to ensure that each item contributes unique information and enhances the scale’s discriminant validity; (iii) explore alternative methods, such as factor analysis, to further evaluate the scale and identify any redundant items within the scale.

## Conclusion

This novel ambulatory teaching program based on the specifications for standardized residency training for outpatient management in general practice presents new recommendations for the ambulatory training of GPs in China. This study suggests selecting patients with multiple comorbidities for ambulatory teaching and enhancing training on practical problem-solving abilities for GPs. The findings provide insights for the development of future ambulatory teaching programs.

### Electronic supplementary material

Below is the link to the electronic supplementary material.

Supplementary Material 1

Supplementary Material 2

Supplementary Material 3

Supplementary Material 4

## Data Availability

The datasets used and/or analyzed during the current study available from the corresponding author on reasonable request.
