# Supplementary material for: A novel model of ambulatory teaching of residents in general practice in China: a cross-sectional study
Source: BMC Med Educ. 2024 Jun 19;24:679. doi: 10.1186/s12909-024-05647-0 (PMC11186264; doi:10.1186/s12909-024-05647-0)
Supplement: Supplementary file 1 — Supplementary Material 1 [file 12909_2024_5647_MOESM1_ESM.docx]

**Supplementary material 1**: Satisfaction scales of ambulatory teaching of residents

| Items |
| --- |
| Participating in the outpatient teaching did not interfere with my daily work |
| I have enough time to practice and learn during time of the outpatient teaching |
| The environment of the consulting room is quite comfortable |
| The nursing support is satisfactory |
| The part of independent reception of patients is very helpful to me |
| The part of supplement and analysis by the preceptor is very helpful to me |
| The part of feedback and summary of the reception process by the preceptor of the outpatient teaching is very helpful to me |
| The number of residents who participated in the outpatient teaching with me is reasonable |
| I received a reasonable number of patients |
| I meet patients with a wide range of ages |
| My patient panel is well balanced between men and women |
| I received about the same proportion of initial and re-visit patients |
| I receive about the same proportion of patients with acute and chronic diseases |
| I treat patients with chronic comorbidities |
| In the outpatient teaching program, I encounter with demands of patients that I have not experienced in the ward |
| I treat patients who impressed me a lot in the outpatient teaching program |
| Preceptor provides a good role model of professional behavior |
| Preceptor allows for autonomy in patient care |
| Preceptor respects my clinical judgment |
| Preceptor is effective at assisting with generating a differential diagnosis |
| Preceptor gives constructive and timely feedback |
| Preceptor teaches history taking skills |
| Preceptor teaches physical exam skills |
| Preceptor is effective at communicating his/her own clinical reasoning processes  when discussing my patients |
| Preceptor is effective at assisting in managing medical issues of my patients |
