# Supplementary material for: A novel model of ambulatory teaching of residents in general practice in China: a cross-sectional study
Source: BMC Med Educ. 2024 Jun 19;24:679. doi: 10.1186/s12909-024-05647-0 (PMC11186264; doi:10.1186/s12909-024-05647-0)
Supplement: Supplementary file 2 — Supplementary Material 2 [file 12909_2024_5647_MOESM2_ESM.docx]

**Supplementary material 1**: The integrity of the structure of the SOAP record

| Index | Not applicable | Content completely missing | Content partially missing | Content complete |
| --- | --- | --- | --- | --- |
| *Subjective section* |  |  |  |  |
| Basic demographic information |  |  |  |  |
| Description of main symptoms |  |  |  |  |
| Simultaneous phenomenon |  |  |  |  |
| Clinically significant negative symptoms |  |  |  |  |
| Disease development and treatment during the process |  |  |  |  |
| Personal history/past medical history/family history |  |  |  |  |
| *Objective section* |  |  |  |  |
| Vital signs |  |  |  |  |
| Heart and lung examination |  |  |  |  |
| Physical examination of key parts and major positive Signs |  |  |  |  |
| Related negative signs |  |  |  |  |
| Auxiliary examination findings supporting to the diagnosis |  |  |  |  |
| Auxiliary examination findings related to differential diagnosis |  |  |  |  |
| *Assessment section* |  |  |  |  |
| Information summary |  |  |  |  |
| Listing of health issues |  |  |  |  |
| Defining characteristics of diagnosis |  |  |  |  |
| Defining characteristics of differential diagnosis |  |  |  |  |
| *Plan section* |  |  |  |  |
| Arrange auxiliary examination according to health issues |  |  |  |  |
| Treatment plan |  |  |  |  |
| Treatment expectation and patient education |  |  |  |  |
| Time of the next follow-up and review index needed |  |  |  |  |
