# Supplementary material for: A novel model of ambulatory teaching of residents in general practice in China: a cross-sectional study
Source: BMC Med Educ. 2024 Jun 19;24:679. doi: 10.1186/s12909-024-05647-0 (PMC11186264; doi:10.1186/s12909-024-05647-0)
Supplement: Supplementary file 3 — Supplementary Material 3 [file 12909_2024_5647_MOESM3_ESM.docx]

**Supplementary material 2**: Training needs of ambulatory teaching of residents

| Items |
| --- |
| Learn the most common chief complaints and symptoms of outpatients of general practice |
| Identifies patients' reasons for consultation |
| Seeks clarification of words used by patients as appropriate |
| Phrases questions simply and clearly |
| Recognizes patients' verbal and non-verbal cues |
| Exhibits well-organized approach to information gathering |
| Considers physical, social and psychological factors as appropriate |
| Makes, when necessary, proper confrontations or compromise |
| Learn the palpation of the superficial lymph node |
| Learn the palpation of the thyroid and breast nodules |
| Quick physical examination of neural system |
| Quick physical examination of shoulder and neck pain |
| Quick physical examination of lower back pain |
| Identification of common skin diseases in outpatient clinics |
| Learn the disease spectrum of outpatients of general practice |
| Learn the most frequently issued auxiliary examinations in the outpatients of general practice |
| Learn the most frequently prescribed medicine and adverse effects of theses medicines of the outpatients of general practice |
| Learn the cautions of issuing diagnosis certificate in outpatient clinic of general practice |
| Learn the most common alternative therapies in outpatient clinic of general practice |
| Learn how to make decisions that are harmless to patients when GPs are temporarily unable to solve patients’ problems |
| Learn how to checks whether patient has understood information of therapies and follow-up arrangements |
| Learn to develop reasonable follow-up plans and achieve consensus for patients |
| Learn how to find appropriate opportunities for health promotion and disease prevention |
| Learn the latest screening methods and progress in diagnosis and treatment of common diseases of outpatient of general practice |
| Learn the latest knowledge of vaccination |
| Learn how to deal with the patient's sleep disorders |
| Learn how to give advice on exercise to outpatients |
| Learn how to give advice on healthy diet to outpatients |
| Learn how to help outpatients quit smoking |
| Learn how to deal with a patient's psychological counseling |
