# Supplementary material for: A novel model of ambulatory teaching of residents in general practice in China: a cross-sectional study
Source: BMC Med Educ. 2024 Jun 19;24:679. doi: 10.1186/s12909-024-05647-0 (PMC11186264; doi:10.1186/s12909-024-05647-0)
Supplement: Supplementary file 4 — Supplementary Material 4 [file 12909_2024_5647_MOESM4_ESM.docx]

**Supplementary material 2**: Overall evaluation of the SOAP record

| Items | Does not meet the requirements | | Meet the requirements | Excellent performance | |
| --- | --- | --- | --- | --- | --- |
| Information gathering: accuracy, integrity, emphasis | 1 | 2 | 3 | 4 | 5 |
| Listing of health issues: reasonable, logical |  |  |  |  |  |
| Diagnosis and treatment plan: problem-based, evidence-based and patient-centered |  |  |  |  |  |
| Organization: efficiency, conform to the SOAP frame |  |  |  |  |  |
| Communication skill: fluency and effective |  |  |  |  |  |
| Professional quality: patient-centered |  |  |  |  |  |
| Overall impression |  | | | | |
